# Spectrum and characterisation of *BRCA1 *and *BRCA2 *deleterious mutations in high-risk Czech patients with breast and/or ovarian cancer

**DOI:** 10.1186/1471-2407-8-140

**Published:** 2008-05-20

**Authors:** Eva Machackova, Lenka Foretova, Mirka Lukesova, Petra Vasickova, Marie Navratilova, Ilse Coene, Hana Pavlu, Veronika Kosinova, Jitka Kuklova, Kathleen Claes

**Affiliations:** 1 Department of Cancer Epidemiology and Genetics, Masaryk Memorial Cancer Institute, Brno, Czech Republic; 2 Centre for Medical Genetics, Ghent University Hospital, Gent, Belgium

## Abstract

**Background:**

The incidence of breast cancer has doubled over the past 20 years in the Czech Republic. Hereditary factors may be a cause of young onset, bilateral breast or ovarian cancer, and familial accumulation of the disease. *BRCA1 *and *BRCA2 *mutations account for an important fraction of hereditary breast and ovarian cancer cases. One thousand and ten unrelated high-risk probands with breast and/or ovarian cancer were analysed for the presence of a *BRCA1 *or *BRCA2 *gene mutation at the Masaryk Memorial Cancer Institute (Czech Republic) during 1999–2006.

**Methods:**

The complete coding sequences and splice sites of both genes were screened, and the presence of large intragenic rearrangements in *BRCA1 *was verified. Putative splice-site variants were analysed at the cDNA level for their potential to alter mRNA splicing.

**Results:**

In 294 unrelated families (29.1% of the 1,010 probands) pathogenic mutations were identified, with 44 different *BRCA1 *mutations and 41 different *BRCA2 *mutations being detected in 204 and 90 unrelated families, respectively. In total, three *BRCA1 *founder mutations (c.5266dupC; c.3700_3704del5; p.Cys61Gly) and two *BRCA2 *founder mutations (c.7913_7917del5; c.8537_8538del2) represent 52% of all detected mutations in Czech high-risk probands. Nine putative splice-site variants were evaluated at the cDNA level. Three splice-site variants in *BRCA1 *(c.302-3C>G; c.4185G>A and c.4675+1G>A) and six splice-site variants in *BRCA2 *(c.475G>A; c.476-2>G; c.7007G>A; c.8755-1G>A; c.9117+2T>A and c.9118-2A>G) were demonstrated to result in aberrant transcripts and are considered as deleterious mutations.

**Conclusion:**

This study represents an evaluation of deleterious genetic variants in the *BRCA1 *and *2 *genes in the Czech population. The classification of several splice-site variants as true pathogenic mutations may prove useful for genetic counselling of families with high risk of breast and ovarian cancer.

## Background

The incidence of breast cancer has doubled over the past 20 years in the Czech Republic [1]. A family history of breast cancer is one of the most significant risk factors for development of the disease. Hereditary factors may cause early onset, bilateral breast or ovarian cancer, and familial accumulation of the disease. Approximately 5 to 10% of all breast cancer cases, and 25 to 40% of cases diagnosed under the age of 35 years, have hereditary origins [2].

Mutations in *BRCA1 *(breast cancer 1, early onset; MIM#113705) and *BRCA2 *(Breast Cancer Type 2 susceptibility protein; MIM#600185) account for an autosomal dominant transmission of susceptibility to breast and ovarian cancer. Among female *BRCA1 *mutation carriers, the lifetime risk of breast cancer has been estimated at between 65 and 85%, and the risk of ovarian cancer between 39 and 63% [3-5]. For *BRCA2 *mutation carriers, the lifetime risk for breast cancer ranges from 45 to 85%, and between 11 and 27% for ovarian cancer [3,5]. The large variations suggest that cancer risk associated with *BRCA *mutations may be modified by other genetic factors in addition to lifestyle and environmental conditions [6].

With the exception of alterations in the extreme carboxy-terminal of *BRCA2 *(3' of the polymorphic stop codon K3326X), all *BRCA1 *and *BRCA2 *truncation mutations are considered to be deleterious [7,8]. Experimental data have revealed the presence of two nuclear localisation signals (NLS) within the final 156 amino acids of the BRCA2 protein. These data indicate that all disease-linked truncating mutations localised upstream of the NLS are likely to be non-functional, losing their ability to translocate to the nucleus [9].

Aside from the many unequivocal pathogenic mutations, several variants of unknown clinical significance have been reported in the *BRCA1 *and *BRCA2 *genes. Messenger ribonucleic acid (mRNA) analysis has only been performed, and the pathogenic effect clearly demonstrated, for a limited number of splice-site alterations [10,11]. In addition, only a few missense mutations have been accepted as deleterious disease-associated genetic alterations that can be reliably used for clinical management. When assessing the clinical significance of a detected variant, several approaches of further analysis are recommended to classify variants as deleterious, suspected deleterious, of unknown (uncertain) clinical significance or polymorphic/neutral [12].

The aim of our study was to demonstrate the pathogenity of all suspected deleterious variants detected in the high-risk Czech population.

## Methods

### Patients and criteria for testing

Patients were referred to the Masaryk Memorial Cancer Institute (MMCI) in Brno for genetic counselling by physicians from various specialisations or were sent for testing by other medical geneticists from various parts of the Czech Republic between 1999 and 2005. All tested individuals provided a signed informed consent following appropriate genetic counselling. Genetic testing was offered to high-risk individuals meeting any of the following criteria:

I. At least 3 diagnoses of breast and/or ovarian cancer in first and/or second degree relatives, diagnosed at any age (320 families).

II. Two affected first degree relatives (or second degree in the case of paternal transmission) diagnosed with breast and/or ovarian cancer, at least one of them diagnosed before the age of 50 (252 families).

III. Any patient with a) bilateral breast, first diagnosed before 50 (29 patients), or b) bilateral ovarian cancer, diagnosed before 50 (7 patients), or c) both breast and ovarian cancer, without an apparent family history of these cancers (19 patients).

IV. Early-onset sporadic unilateral breast (121 patients) or ovarian (19 patients) cancer cases diagnosed before the age of 40.

V. Any male patient with breast cancer, independent of age at diagnosis and family history (16 males who do not fulfil criteria I or II).

VI. In several families with hereditary breast and/or ovarian cancer predisposition, none of the affected relatives was available for testing. Healthy high-risk individuals, who had a 50% chance of being a carrier of the familial mutation, were therefore analysed (77 families).

VII. In addition, we analysed 150 affected probands on the borderline of our inclusion criteria (such as probands from small families with 2 affected relatives diagnosed between 51 to 60 years of age, or patients diagnosed before the age of 50 with a third degree relative with breast/ovarian cancer) referred by clinical geneticists.

Inclusion criteria I, II and VI were considered as familial cases, whilst criteria III, IV and V were considered as severe sporadic cases.

### Mutation screening

Genomic DNA was isolated from blood samples with the QIAamp DNA blood purification kit (Qiagen). The non-radioactive protein truncation test (PTT) (Promega) was used for analysis of exon 11 of the *BRCA1 *gene, and for exons 10 and 11 of the *BRCA2 *gene. The remaining exons and their splice sites were screened by Heteroduplex analysis (HA) using Mutation Detection Enhancement (MDE) gel solution (Cambrex), essentially as described by Claes *et al*. [13]. Fragments with aberrant mobility, detected on PTT or HA gels, were sequenced on an independently amplified polymerase chain reaction (PCR) product by direct sequencing using the Thermo Sequenase Primer Cycle Sequencing Kit, or the Thermo Sequenase Cy5 Dye Terminator Cycle Sequencing Kit (Amersham Biosciences), and analysed on the ALF express™ DNA sequencer (Pharmacia). Each newly found sequence alteration was confirmed by sequencing from both forward and reverse directions. Each detected mutation was confirmed on an independently drawn blood sample. In the case of a deceased patient, a germline mutation was confirmed on DNA isolated from non-tumour paraffin embedded tissue.

In addition, we evaluated the frequency of large genomic rearrangements in the *BRCA1 *gene with Multiplex Ligation-dependent Probe Amplification, or MLPA (MRC-Holland). More details about this study are reported elsewhere [14].

### cDNA analysis

All putative splice-site variants were tested by the Splice Site Prediction Programs for their potential to alter splicing [11]. Subsequently, complementary DNA (cDNA) analysis was performed to verify the *in silico *results.

Total ribonucleic acid (RNA), treated with a nonsense-mediated mRNA decay inhibitor (Puromycin), was extracted from short-term PHA-stimulated lymphocyte cultures [15] of the patient and from controls not carrying the studied splice-site alteration.

The region of the *BRCA1*/*BRCA2 *cDNA encompassing the relevant fragment was amplified by PCR. If more than two aberrant transcripts were obtained, the PCR products derived from cDNA templates were cloned into the vector PCR 2.1-TOPO and transfected into Escherichia coli strain Top10F (Invitrogen). After amplification with M13 primers, the products were resolved on agarose gels and sequenced. The cDNA analysis approach has previously been described in more detail by Claes *et al*. [11].

### Nomenclature

Detected mutations and other sequence alterations are described at the cDNA level according to the GenBank: U14680* BRCA1 *reference sequence and GenBank: NM_000059* BRCA2 *reference sequence, using the recommended nomenclature system for human gene mutations [16].

The cDNA numbering for the traditional mutation nomenclature used in the BIC (Breast Cancer Information Core) Database is based on reference sequences as stated above, where the A of the ATG translation initiation codon is at position 120 of the *BRCA1 *mRNA and at position 229 of the *BRCA2 *mRNA, respectively. The HUGO-approved systematic nomenclature follows the rule where the nucleotide +1 is the A of the ATG translation initiation codon. In the text of this article, we use the systematic nomenclature. In the tables, both BIC and systematic cDNA numbering are used.

## Results

### Deleterious BRCA mutations

In this study, we describe molecular genetic analyses of the *BRCA1 *and *BRCA2 *genes in 1,010 unrelated high-risk probands. Through PCR based screening methods, 188 families were identified as carrying deleterious *BRCA1 *mutations (34 different mutations – Table 1) and 80 families as harbouring deleterious *BRCA2 *mutations (35 different mutations – Table 2). Furthermore, we identified 10 different alterations in highly conserved splice sites in 15 families (Table 3), which were analysed at the mRNA level. In 11 additional families, 6 different large intragenic rearrangements in the *BRCA1 *gene were detected by MLPA. These were further characterised and are described in detail elsewhere [14]. Altogether, a pathogenic mutation was found in 294 of 1,010 (29.1%) unrelated families (Tables 1 to 3).

The spectrum of the *BRCA1 *mutations is summarised in Tables 1 and 3. As generally reported, the majority of the *BRCA1 *mutations were mutations leading to a premature stop codon (21 different frame-shift, 9 different nonsense, and 4 different splice-site mutations (described below). Furthermore, we identified 4 different deleterious missense mutations localised in the 100% conserved cysteine residues of the *BRCA1 *C_3_HC_4 _RING domain: p.Cys24Tyr; p.Cys39Arg; p.Cys61Gly and p.Cys64Tyr.

**Table 1:** Spectrum of frame-shift, missense and nonsense *BRCA1 *germline mutations detected in Czech patients.

*BRCA1 *exon	DNA level BIC traditional nomenclature	DNA level Systematic nomencl.	Protein level	No. of families
2	c.174C>T	c.55C>T	p.Gln19X	1
2	c.187_188delAG (185delAG)	c.68_69del2	p.Glu23ValfsX17	7
2	c.190G>A	c.71G>A	p.Cys24Tyr	1
3	c.234T>C	c.115T>C	p.Cys39Arg	5
5	c.300T>G	c.181T>G	p.Cys61Gly	20
5	c.310G>A	c.191G>A	p.Cys64Tyr	1
11A	c.962_965del4	c.843_846del4	p.Ser282TyrfsX15	1
11A	c.1135dupA (1135insA)	c.1016dupA	p.Val340GlyfsX6	1
**11A**	**c.1159delT**	**c.1040delT**	**p.Leu347ArgfsX27**	**1**
**11A**	**c.1187_1196del10**	**c.1068_1077del10**	**p.Gln356HisfsX15**	**1**
11A	c.1323delG	c.1204delG	p.Glu402SerfsX8	1
**11A**	**c.1522delA**	**c.1403delA**	**p.Lys468ArgfsX7**	**1**
11A	c.1629delC	c.1510delC	p.Arg504ValfsX28	1
**11A**	**c.1719C>T**	**c.1600C>T**	**p.Gln534X**	**3**
11A	c.1806C>T	c.1687C>T	p.Gln563X	6
**11B**	**c.2276dupA**	**c.2157dupA**	**p.Glu720ArgfsX6**	**2**
**11B**	**c.2312_2315del4**	**c. 2193_2196del4**	**p.Glu732ArgfsX3**	**1**
11B	c.2382G>T	c.2263G>T	p.Glu755X	4
11B	c.2530_2531delAG	c.2411_2412del2	p.Gln804LeufsX5	3
**11B**	**c.2607_2616dup10 **(2616_2617ins10)	**c.2488_2497dup10**	**p.Leu833X**	**4**
**11B**	**c.2881delA**	**c.2762delA**	**p.Gln921ArgfsX79**	**2**
**11B**	**c.3283delG**	**c.3164delG**	**p.Gly1055AlafsX4**	**1**
**11C**	**c.3740_3745del6insAA**	**c.3591_3626del6insAA**	**p.Leu1209Ser25**	**1**
**11C**	**c.3761_3762delGA**	**c.3642_3643del2**	**p.Asn1215fsX3**	**2**
11C	c.3819_3823del5	c.3700_3704del5	p.Val1234GlnfsX8	29
11C	c.3875_3878del4	c.3756_3759del4	p.Ser1253ArgfsX10	6
**11C**	**c.4171T>A**	**c.4052T>A**	**p.Leu1351X**	**1**
12	c.4280_4281delTC	c.4161_4162del2	p.Gln1388GlufsX2	1
12	c.4284_4285delAG	c.4165_4166del2	p.Ser1389X	1
13	c.4458C>T	c.4339C>T	p.Gln1447X	1
20	c.5385dupC (5385insC; 5382insC)	c.5266dupC	p.Gln1756ProfsX74	75
22	c.5465G>A	c.5346G>A	p.Trp1782X	1
24	c.5629G>A	c.5510G>A	p.Trp1837X	1
24	c.5630G>A	c.5511G>A	p.Trp1837X	1

*BRCA1 *reference sequence GenBank: U14680. Mutations marked in bold had not been submitted to the BIC database from any other population prior to November 2007 [21].

**Table 2:** Spectrum of frame-shift and nonsense *BRCA2 *germline mutations detected in Czech patients.

*BRCA2 *exon	DNA level BIC traditional nomenclature	DNA level Systematic nomencl.	Protein level	No. of families
**3**	**c.534dupA**	**c.306dupA**	**p.Leu103IlefsX10**	**1**
5	c.690_691delAA	c.462_463del2	p.Asp156X	1
9	c.983_986del4	c.755_758del4	p.Asp252ValfsX24	1
10	c.1617_1618delAG	c.1389_1390del2	p.Val464GlyfsX3	2
10	c.2024_2028del5	c.1796_1800del5	p.Phe559X	1
10	c.2041dupA (2041–2042insA)	c.1813dupA	p.Ile605AsnfsX11	2
11A	c.3036_3039del4	c.2808_2811del4	p.Ala938ProfsX21	5
11A	c.3304A>T	c.3076A>T	p.Lys1026X	1
**11A**	**c.3337C>T**	**c.3109C>T**	**p.Gln1037X**	**1**
11B	c.3972_3975del4	c.3744_3747del4	p.Ser1248ArgfsX10	1
11B	c.4075_4076delGT	c.3847_3848del2	p.Val1283LysfsX2	2
**11C**	**c.5073_5074delCT**	**c.4845_4846del2**	**p.Leu1616LysfsX2**	**1**
11C	c.5270_5271delGT	c.5042_c.5043del2	p.Val1681GlufsX7	1
11D	c.5441_5444del4	c.5213_5216del4	p.Thr1738IlefsX2	1
**11D**	**c.5869_5872del4**	**c.5641_5644del4**	**p.Lys1881GlnfsX27**	**1**
11D	c.5873C>A	c.5645C>A	p.Ser1882X	4
11D	c.5950_5951delCT	c.5722_5723del2	p.Leu1908ArgfsX2	1
11D	c.5991dupT	c.5763dupT	p.Ala1922CysfsX2	1
**11D**	**c.6017delT**	**c.5789delT**	**p.Leu1930TyrfsX33**	**1**
11E	c.6633_6637del5	c.6405_6409del5	p.Asn2135LysfsX3	1
**11E**	**c.6677_6678delAA**	**c.6449_6450del2**	**p.Lys2150SerfsX25**	**3**
11E	c.6696_6697delTC	c.6468_6469del2	p.Gln2157IlefsX18	1
**11E**	**c.6866delC**	**c.6638delC**	**p.Ser2213X**	**2**
**11E**	**c.6982dupT (6982–6983insT)**	**c.6754dupT**	**p.Ser2252PhefsX9**	**1**
**14**	**c.7379_7380delAA**	**c.7151_7152del2**	**p.Gln2384ArgfsX7**	**1**
15	c.7699C>T	c.7471C>T	p.Gln2491X	1
17	c.8141_8145del5 (8138_8142del5)	c.7913_7917del5	p.Phe2638fsX	15
**18**	**c.8270_8271delCA**	**c.8042_8043del2**	**p.Thr2681CysfsX11**	**3**
**18**	**c.8397_8400dup4 (8400_8401ins4)**	**c.8169_8172dup4**	**p.Trp2725MetfsX3**	**1**
**19**	**c.8591G>A**	**c.8363G>A**	**p.Trp2788X**	**1**
20	c.8765_8766delAG	c.8537_8538del2	p.Glu2846GlyfsX22	14
23	c.9325dupA	c.9097dupA	p.Thr3033AsnfsX11	1
25	c.9631delC	c.9403delC	p.Leu3135PhefsX28	4
25	c.9663_9664delGT	c.9435_9436del2	p.Ser3147CysfsX2	1
**25**	**c.9691_9695del5ins8**	**c.9463_9467del5ins8**	**p.Phe3155GlufsX9**	**1**

*BRCA2 *reference sequence GenBank: U43746. Mutations marked in bold had not been submitted to the BIC database from any other population prior to November 2007 [21].

**Table 3:** Splice-site alterations detected in Czech high-risk breast/ovarian cancer patients.

**DNA level Gene**/splice site BIC/**systematic **nomenclature	Predicted consensus values*		**RNA level **(systematic nomencl.) experimentally determined	**Protein level **deduced	**Criteria **of affected probands	**Comment**
					
	***acceptor***	***donor***				
**BRCA1**/IVS6, wild type	0,99		r.302-2_302-1ins	p.Tyr101X	III (bi-brca 35,55)	
c. 421-3C>G/**c.302-3C>G**	**new 0,9 **at position -2					

**BRCA1**/exon 12, wild type		0,95	r.4097_4185del	p.Gly1366AlafsX8	I (HBC)	Cfr. Ref. [11]
c.4304G>A/**c.4185G>A**		**0,36**				

**BRCA1**/IVS15, wild type		0,44	r. [=, 4358_4675del, 4485_4675del, 4665_4675del, 4358_4484del+4665_4675del]	p. [=, Ala1453_Leu1558del, Ser1496GlyfsX14, Gln1556GlyfsX14, Ala1453GlyfsX10]	VII (brca 42)	
c.4794+1G>A/**c.4675+1G>A**		**not predicted**				

**BRCA1**/IVS18, wild type		0,95	*Not analysed; all known mutation carriers were deceased*	p.?	II (HBC)	Highly suspected to be deleterious.
c.5271+2dupT/**c.5152+2dupT**		**not predicted**				

**BRCA2**/exon 5, wild type		0,95	r.326_475del	p.Phe143GlyfsX26	I (HBC)	
c.703G>A/**c.475G>A**		**0,46**			VI (HBC-FA-I)	

**BRCA2**/IVS5, wild type	0,91		r. [326_516del, 476_516del]	p. [Phe143ValfsX12, Val159GlyfsX10]	I (HOC); II (HBC)	
c.704-2A>G/**c.476-2A>G**	**not predicted**				IV (brca 27)	
					IV(brca 34)	

**BRCA2**/exon13, wild type		0,99	r. [6842_6937del, 6842_7007del, 6938_7007del]	p. [Glu2281_Gly2313del, Gly2280AlafsX31, Gly2313Ala fsX31]	I (HBC)	Cfr. Ref. [25]
c.7235G>A/**c.7007G>A**		**not predicted**			V(male brca)	

**BRCA2**/IVS21, wild type	not predicted		r.8755_8953del	p.Gly2919fsX3	II (HBC)	
c.8983-1G>A/**c.8755-1G>A**	**not predicted**					

**BRCA2**/IVS23, wild type		0,57	r.8954_9117del	p.Val2985GlyfsX4	I (HBOC)	
c.9345+2T>A/**c.9117+2T>A**		**not predicted**				

**BRCA2**/IVS23, wild type	1,00		r.9118_9124del	p.Val3040MetfsX20	I (HBC)	Cfr. Ref. [11]
c.9346-2A>G/**c.9118-2A>G**	**cryptic 0,7**					

* Predicted consensus values by the Splice Site Prediction Program: Neural Networks [23].

All deleterious mutations identified in the *BRCA2 *gene were truncating mutations (30 different frame-shift mutations, 5 different nonsense mutations, and 6 different splice-site mutations (described below)) and are summarised in Tables 2 and 3.

### Splice-site alterations

For all putative splice-site variants, we performed further analyses to unequivocally determine their pathogenic effect. We have previously reported that *BRCA1 *c.4185G>A and *BRCA2 *c.9118A-2>G lead to aberrant transcripts [11]. In this publication, we provide results of the effects at the cDNA level for 7 additional putative splice-site variants that have not been described previously, i.e. two variants in the *BRCA1 *gene (c.302-3C>G and c.4675+1G>A) and five in the *BRCA2 *gene (c.475G>A; c.476-2>G; c.7007G>A; c.8755-1G>A and c.9117+2T>A).

To set aside nonsense-mediated mRNA decay (NMD), an inhibitor of the NMD pathway was used before RNA isolation from lymphocyte cultures. As we had previously found that splice-site mutations not only lead to transcripts affecting the exon following or preceding the mutation but may also affect other neighbouring exons, we chose RT-PCR primers that were not in the immediately adjacent exons.

**Figure 1:**
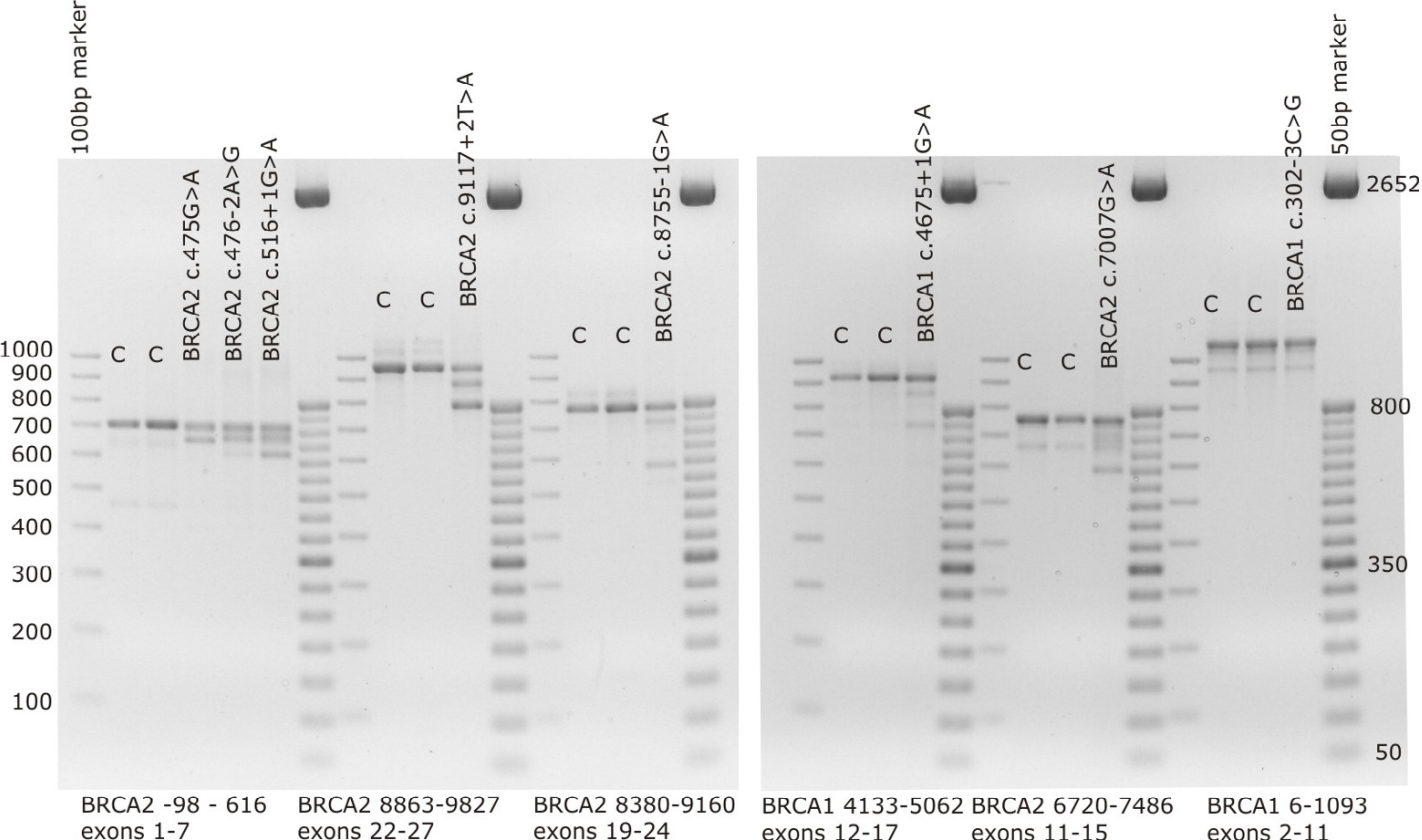
**RT-PCR results for the splice-site mutations identified in our patient population.** Below the picture we indicate the position of the RT primers used. C = control. A detailed description of the relevant fragments obtained can be found in Table 3. However, we omitted in this Table details about several novel alternative transcripts containing intronic sequences with high acceptor/donor scores at the 5'/3' ends, complicating the interpretation of the results and of which the clinical relevance is currently unknown. For instance, cloning RT-PCR products spanning exons 21 to 26 revealed several novel alternative transcripts containing insertions from sequences of introns 24 and 25 (cfr. extra bands above the full length fragment in control persons and bands around 920–930 bp in the patient with *BRCA2 *c.9117+2T>A, representing transcripts lacking exon 23 but containing these intronic sequences. Similar observations were obtained for the RT-PCR analyses for *BRCA2 *c.8755-1G>A.

Results of the RT-PCR experiments are shown in Figure 1. The predicted consensus values, the experimentally determined effect on mRNA, and deduced results at the protein level are summarised in Table 3. For 4 of the putative splice-site mutations analysed (*BRCA1 *c.4675+1G>A, *BRCA2 *c.476-2A>G, c.8755-1G>A and c.9117+2T>A), aberrant splicing was anticipated as these variants affect the highly conserved nucleotides at positions +1+2/-1-2 of the splice donor/acceptor sites.

Furthermore, we validated 3 variants outside the highly conserved +1+2/-1–2 nucleotides. *BRCA1 *c.302-3C>G activates a cryptic splice site in intron 6, leading to the inclusion of two extra base pairs from this intron. This results in a frame-shift and a premature termination of translation.

The G to A transition in the last nucleotide of *BRCA2 *exon 5 (c.475G>A) results in a transcript lacking *in frame *exon 5, which was absent in control individuals. Unfortunately, no patient with both this mutation and an SNP in exons 2–10 of *BRCA2 *was available to allow investigation of whether the mutant allele was still able to transcribe a full- length product. This mutation was identified in two unrelated families that were too small to evaluate segregation.

RT-PCR with a forward primer located in exon 11 and a reverse primer in exon 15 revealed that the *BRCA2 *c.7007G>A mutation, in the last nucleotide of exon 13, is not a missense mutation (p.Arg2336His). We found a transcript lacking exon 13, a transcript lacking exons 12 and 13, and a transcript lacking exon 12. The transcript lacking exon 12 is present at about the same level in blood lymphocytes of both the patient with the mutation and a control person (data of the cloning experiments not shown). The *BRCA2 *c.7007G>A mutation was found in 2 unrelated families: in an affected female with a severe family history of breast cancer, fulfilling criterion I, and in an affected male with a maternal aunt and cousin diagnosed with breast cancer.

### Mutation detection rates

*BRCA1 *mutations were found to be about 2 times more frequent than *BRCA2 *mutations in our study population. Mutation detection frequencies (deleterious and splice-site mutations and large intragenic rearrangements) in relation to the inclusion criteria are summarised in Table 4. Surprisingly, the highest mutation frequency (14 out of 19 cases, or 73.7%) was obtained in apparently sporadic patients diagnosed with both breast and ovarian cancers. By re-evaluation of the pedigrees, we found that the apparent lack of a family history might have been due to a small number of relatives and/or paternal transmission in these families. As expected, a higher mutation detection rate was obtained in breast and ovarian (HBOC) cancer families fulfilling criteria I and II (96/160, or 60%) as compared to breast cancer only (HBC) families fulfilling the same criteria (114/412, or 27.7%).

**Table 4:** Summary of deleterious mutations in *BRCA *genes identified in relation to inclusion criteria and phenotype.

Inclusion criteria and phenotype	Number of families/patients	**BRCA1 **mutation N (%; 95% CI)	**BRCA2 **mutation N (%; 95% CI)	Overall **mutation **N (%; 95% CI)
I. HOC + HBOC	120	61 (50.8%; 41.6%–60.1%)	12 (10.0%; 5.3%–16.8%)	73 (60.8%; 51.5%–69.6%)
I. HBC	200	41 (20.5%; 15.1%–26.8%)	24 (12.0%; 7.8%–17.3%)	65 (32.5%; 26.1%–39.5%)
I. Overall	320	102 (31.9%; 26.8%–37.3%)	36 (11.3%; 8.0%–15.2%)	138 (43.1%; 37.6%–48.8%)
				
II. HOC + HBOC	40	22 (55.0%; 38.5%–70.7%)	1 (2.6%; 0.1%–13.2%)	23 (57.5%; 40.9%–73.0%)
II. HBC	212	25 (11.8%; 7.8%–16.9%)	24 (11.3%; 7.4%–16.4%)	49 (23.1%; 17.6%–29.4%)
**II. Overall**	252	47 (18.7%; 14.0%–24.0%)	25 (10.0%; 6.5%–14.3%)	72 (28.6%; 23.1%–34.6%)
				
I.+II. Familial cases HOC + HBOC	160	83 (51.9%; 43.8%–59.8%)	13 (8.2%; 4.4%–13.5%)	96 (60.0%; 52.0%–67.7%)
I.+II. Familial cases HBC	412	66 (16.0%; 12.6%–19.9%)	48 (11.7%; 8.7%–15.1%)	114 (27.7%; 23.4%–32.3%)
**I.+II. Familial cases – overall**	572	149 (26.0%; 22.5%–29.9%)	61 (10.7%; 8.3%–13.5%)	210 (36.7%; 32.8%–40.8%)
				
III.A Bilateral breast cancer patient	29	5 (17.2%; 5.8%–35.8%)	4 (13.8%; 3.9%–31.7%)	9 (31.0%; 15.3%–50.8%)
III.B Bilateral ovarian cancer patient	7	1 (14.3%; 0.4%–57.9%)	0 (0.0%; 0.0%–0.0%)	1 (14.3%; 0.4%–57.9%)
III.C Patient with breast and ovarian cancer	19	11 (57.9%; 33.5%–79.7%)	3 (15.8%; 3.4%–39.6%)	14 (73.7%; 48.8%–90.9%)
**III. Duplex cancer patients -overall**	55	17 (30.9%; 19.1%–44.8%)	7 (12.7%; 5.3%–24.5%)	24 (43.6%; 30.3%–57.7%)
				
IV. Early onset ovarian cancer patient	19	1 (5.3%; 0.1%–26.0%)	0 (0.0%; 0.0%–0.0%)	1 (5.3%; 0.1%–26.0%)
IV. Early onset breast cancer patient	121	7 (5.8%; 2.4%–11.6%)	6 (5.0%; 1.8%–10.5%)	13 (10.7%; 5.8%–17.7%)
**IV. Overall**	140	8 (5.7%; 2.5%–10.9%)	6 (4.3%; 1.6%–9.1%)	14 (10.0%; 5.6%–16.2%)
				
V. Male breast cancer	16	3 (18.8%; 4.0%–45.6%)	3 (18.8%; 4.0%–45.6%)	6 (37.5%; 15.2%–64.6%)
VI. Healthy person in high-risk (I.) family	77	15 (19.5%; 11.3%–30.1%)	5 (6.5%; 2.1%–14.5%)	20 (26.0%; 16.6%–37.2%)
VII. Out of criteria families	150	12 (8.0%; 4.2%–13.6%)	8 (5.3%; 2.3%–10.2%)	20 (13.3%; 8.3%–19.8%)

*Abbreviations: *inclusion criteria – see Methods section; mutation – only deleterious mutations summarised in Table 1 and Table 2 are considered; CI – Confidence Interval; HOC – hereditary ovarian cancer syndrome; HBOC – hereditary breast and ovarian cancer syndrome; HBC – hereditary breast cancer only syndrome.

Six out of 16 male breast cancer patients (37.5%) carried a deleterious mutation. Unexpectedly, the same number of mutations was identified in the *BRCA2 *gene (c.7913_7917del5; c.9403delC, and the splice-site mutation c.7007G>A) as in the *BRCA1 *gene (2 males carrying c.5266dupC and one patient with c.3700_3704del5). The average age of male breast cancer diagnosis in mutation carriers was 72.5 years (ranging from 58 to 80 years). Four of the mutation carriers reported a family history of breast or prostate cancer but did not meet inclusion criteria II. The average age at diagnosis of breast cancer of the 10 males without a *BRCA *mutation was 57.2 years, ranging from 37 to 80 years. In addition, we identified rare variants of unknown significance in four male patients: 2 in *BRCA1 *(c.5277+48_5277+59dup12; c. 5277+78G>A) and 2 in the *BRCA2 *gene (p.Ala75Pro; c.10095delCins11).

A deleterious mutation was detected in 20 out of 77 healthy, unaffected probands (26%; 15 in *BRCA1 *and 5 in the *BRCA2 *gene) with a severe family history of breast and/or ovarian cancer but no living affected relative. Furthermore, we identified a deleterious mutation in 20 out of 150 affected probands (13.3%; 12 in *BRCA1 *and 8 in the *BRCA2 *gene) who did not fully meet our inclusion criteria for testing but who were referred by genetic counsellors.

## Discussion

Molecular genetic analysis for the *BRCA1 *and *BRCA2 *genes has been available at the MMCI since 1999. By the end of 2006, an unequivocal deleterious mutation had been identified in 294 high-risk Czech probands out of 1,010 probands selected for genetic testing (29.1%). Similar inclusion criteria are used in other centres [17].

In familial cases, we obtained a mutation detection rate of 36.7% (210/572) in sporadic bilateral breast, bilateral ovarian or breast, and ovarian cancer patients 43.6% (24/55) but only 10.7% (13/121) in early onset unilateral breast cancer patients. However, the latter is slightly higher than generally reported in Europe (< 5–9%) in early onset sporadic cases [18,17]. *BRCA1 *mutations were twice as frequent as *BRCA2 *mutations in our study population. The presence of an ovarian cancer case turned out to be a good indicator for the presence of a causative *BRCA *mutation: the highest mutation detection rates were obtained in apparently sporadic patients diagnosed with both breast and ovarian cancer (73,7%) and in breast-ovarian cancer families (about 60%). This detection rate seems to be rather high when compared with ones reported for example in Germany (43%) or Belgium using similar inclusion criteria [18,17]. Detection rate highly depends on severity of family history and this might be result of accumulating of very severe affected families seeking genetic advice in our population.

The majority of the *BRCA1 *and *2 *deleterious mutations lead to a premature termination of translation. As the protein truncation test was used for screening *BRCA1 *exon 11 and *BRCA2 *exons 10 and 11, missense mutations were not detectable in these exons. To date, however, no undoubtedly deleterious missense mutations have been reported for these exons. Truncating mutations may be assumed to cause disease, but the pathogenic effect of missense and intronic variants is more equivocal. Only the p.Cys24Tyr; p.Cys39Arg; p.Cys61Gly and p.Cys64Tyr missense mutations in the *BRCA1 *gene are classified as deleterious mutations. These substitutions take place in the 100% conserved cysteine residues of the *BRCA1 *C_3_HC_4 _RING domain. All these missense mutations in the Zn^2+^-binding residues of the C_3_HC_4 _RING domain have previously been experimentally shown to abolish dimerization of *BRCA1 *with the BARD1 protein, resulting in loss of ubiquitin protein ligase activity and causing γ-radiation hypersensitivity [19,20]. The p.Cys61Gly is one of the most frequent founder *BRCA1 *mutations found in the Czech Republic, as well as in many other European countries [21].

Five mutations (*BRCA1: *p.Cys61Gly, c.5266dupC, c.3700_3704del5, and *BRCA2: *c.7913_7917del5 and c.8537_8538delAG) represent 52% of all mutations detected in our study population. Although there was a diversity of mutations in both genes, there are strong Slavic founder effects, particularly for two *BRCA1 *mutations (p.Cys61Gly, and c.5266dupC) found as founder mutations in Poland [21,22]. The *BRCA1 *c.3700_3704del5 mutation is a frequent mutation in Germany [17,21]. Three *BRCA1 *Czech founder mutations (p.Cys61Gly, c.3700_3704del5, and c.5266dupC) accounted for 9.8%, 14.2% and 36.8% of the *BRCA1 *mutations identified, respectively. A Czech founder effect is evident for two *BRCA2 *mutations (c.7913_7917del5 and c.8537_8538del2) that are reported elsewhere only from Canada and the USA [21]. These *BRCA2 *mutations accounted for 15.6% and 16.7% of the *BRCA2 *mutations identified, respectively.

A broad spectrum of other mutations demonstrates the genetic diversity of the Czech population and reflects the spectrum of mutations found in other European countries [21]. The Czech population is mainly of Slavic origin. Due to its location in Central Europe, however, and historical influences during past centuries, when the Czech region was a part of the Austro-Hungarian Empire, an influx of other nations is evident.

All putative splice-site mutations were tested computationally for their ability to change the donor/acceptor score *in silico*, as well as to elucidate the creation or activation of cryptic splice sites. For all except one (*BRCA1 *c.4675+1G>A), the novel splice sites were correctly identified by the Neural Networks prediction program, thereby demonstrating its usefulness [23].

For several variants (*BRCA1: *c.302-3C>G; c.4675+1G>A and *BRCA2 *c.475G>A, c.476-2>G; c.7007G>A; c.8755-1G>A; c.9117+2T>A), reliable RT-PCR data were not available from the literature or the BIC database. For genetic counselling purposes, it is extremely important to differentiate between deleterious and polymorphic splice-site alterations. Analysis at the mRNA level allowed us to identify alterations leading to aberrant splicing. Especially for genetic alterations outside the almost 100% conserved +1/+2 and -1/-2 nucleotides, RT-PCR analyses can contribute to a correct and definitive clinical interpretation. We clearly demonstrate here that *BRCA1 *c.302-3C>G and *BRCA2 *c.475G>A, c.7007G>A lead to aberrant splicing.

In general, splice-site alterations lead to skipping of the complete exon following the acceptor or preceding the donor site in which the mutation is located. This was indeed what we found for *BRCA1 *c.4185G>A and *BRCA2 *c.475G>A; c.9117+2T>A and c.8755-1G>A. Other mutations result in the creation of a novel splice site or activation of a cryptic splice site, such as was found for *BRCA1 *c.302-3C>G and *BRCA2 *c.9118-2A>G.

Mutations in splice sites of some exons lead to multiple exon skipping. For example, mutations in the splice sites of *BRCA2 *exon 6 seem to also affect exon 5, as observed from RT-PCR analyses for c.476-2A>G and the previously reported c.516+1G>A mutation [[11], Figure 1]. A further example is the *BRCA1 *c.4675+1G>A mutation in intron 15. mRNA analysis revealed several different transcripts, including some with a skip of exon 14. Multiple exon skipping has previously been shown to be associated with mutations in 5' as well as 3' splice sites in several genes [24]. By the identification and further functional analyses of such splice mutations, new regulatory elements and mechanisms involved in splicing may be revealed.

The *BRCA2 *c.7007G>A mutation, in the last nucleotide of exon 13, was previously studied by RT-PCR with primers located in exons 12 and 14, revealing only a transcript lacking exon 13 [25]. As we chose RT-PCR primers that were not in the immediately adjacent exons, we discovered a more complex splicing pattern, i.e. a transcript lacking exon 13, a transcript lacking exons 12 and 13, and a transcript lacking exon 12. The latter transcript was present at the same level in control persons. An exon 12 alternatively spliced *BRCA2 *transcript has previously been described as a major alternative *BRCA2 *transcript with an expression level of about 10% in normal breast tissue relative to the wild type *BRCA2 *transcript. The expression level of the delta12-*BRCA2 *transcript has been shown to be higher in some tumour tissues compared with their normal breast tissues [26].

In addition, our cDNA analyses revealed several novel alternative transcripts containing intronic sequences with high acceptor/donor scores at the 5'/3' ends. Further investigations are required to determine the prevalence of these transcripts in different tissues and to define their possible role. A limitation of our studies is that they were performed on transcriptional products of peripheral lymphocytes, rather than on the breast or ovarian tissues that are considered to be the sites of initial abnormalities in *BRCA1/2*-related tumors. Further studies are needed to assess whether different quantitative and/or qualitative effects of the alterations investigated are observed in these tissues.

Numerous *BRCA1 *and *BRCA2 *sequence alterations identified during screening are kept in the laboratory database. Nonsynonymous single nucleotide polymorphisms still may have the potential to represent modifiers of inherited susceptibility. As deleterious germline mutations in *BRCA1 *and *BRCA2 *confer a greatly increased risk of breast cancer, some sequence variants may be only moderate or low penetrant alleles.

## Conclusion

We detected 204 families harbouring deleterious *BRCA1 *mutations (44 different), and 90 families harbouring *BRCA2 *mutations (41 different). *BRCA1 *mutations were found to be about 2 times more frequent than *BRCA2 *mutations in high-risk members of the Czech population, based on our study population. Mutation detection frequencies (deleterious and splice-site mutations and large intragenic rearrangements) in relation to the inclusion criteria were highest in the group of patients diagnosed with both breast and ovarian cancers (73.7%) and in familial cases with clustering of breast and ovarian cancers in first and second degree relatives (60%).

Five founder mutations, three *BRCA1 *(c.5266dupC; c.3700_3704del5; p.Cys61Gly) and two *BRCA2 *mutations (c.7913_7917del5; c.8537_8538del2), account for 52% of all detected mutations. These mutations could be used as a first-step screen. However, the screening of the whole region of both genes is highly indicated for high-risk Czech breast/ovarian cancer patients.

We evaluated 9 putative splice-site variants at the cDNA level. Three splice-site variants in the *BRCA1 *gene (c.302-3C>G; 4185G>A and c.4675+1G>A) and six splice-site variants in the *BRCA2 *gene (c.475G>A; c.476-2>G; c.7007G>A; c.8755-1G>A; c.9117+2T>A and c.9118-2A>G) were demonstrated to result in aberrant transcripts and are considered as deleterious mutations. As deleterious germline mutations in *BRCA1 *and *BRCA2 *confer a greatly increased risk of breast cancer, some sequence variants in both genes might be candidates for moderate or low penetrance alleles. Each sequence variant detected in high-risk probands, therefore, has to be evaluated through a complex approach to establish the clinical consequence. A result obtained through genetic testing for hereditary breast and ovarian cancer has great impact on the medical management of a patient and accurate interpretation is, therefore, imperative.

## Abbreviations

**CI **– Confidence Interval; **HA **– Heteroduplex Analysis; **HBC **– Hereditary Breast Cancer; **HBOC **– Hereditary Breast and Ovarian Cancer; **HOC **– Hereditary Ovarian Cancer; **MMCI **– Masaryk Memorial Cancer Institute; **NLS **– Nuclear Localization Signal; **NMD **– Nonsense-mediated mRNA Decay; **PCR **– Polymerase Chain Reaction; **PTT **– Protein Truncation Test.

## Competing interests

The authors declare that they have no competing interests.

## Authors' contributions

EM, ML and LF participated equally in the design of this study. ML, EM and PV carried out the molecular analysis and interpreted the results. LF and MN participated in genetic counselling and the selection of participating patients and their family members. KC and IC provided the mRNA analysis of all putative splice-site variants and their interpretation. HP, VK and JK participated in mutation screening. EM drafted the paper and finalised the manuscript, with the help of KC. All authors read and approved the final manuscript.

## Pre-publication history

The pre-publication history for this paper can be accessed here:


